# Environmental and Anthropogenic Factors Shape the Snow Microbiome and Antibiotic Resistome

**DOI:** 10.3389/fmicb.2022.918622

**Published:** 2022-06-16

**Authors:** Concepcion Sanchez-Cid, Christoph Keuschnig, Karol Torzewski, Łukasz Stachnik, Daniel Kępski, Bartłomiej Luks, Adam Nawrot, Przemysław Niedzielski, Timothy M. Vogel, Catherine Larose

**Affiliations:** ^1^Environmental Microbial Genomics, CNRS UMR 5005 Laboratoire Ampère, École Centrale de Lyon, Université de Lyon, Écully, France; ^2^Promega France, Charbonnières-les-Bains, France; ^3^Department of Ecology, Biogeochemistry and Environmental Protection, Institute of Botany, Wrocław University, Wrocław, Poland; ^4^Institute of Geography and Regional Development, Faculty of Earth Sciences and Environmental Management, University of Wrocław, Wrocław, Poland; ^5^Institute of Geophysics, Polish Academy of Sciences, Warsaw, Poland; ^6^forScience Foundation, Toruń, Poland; ^7^Department of Analytical Chemistry, Faculty of Chemistry, Adam Mickiewicz University in Poznań, Poznań, Poland

**Keywords:** antibiotic resistance, human activities, snow microbiome, metagenomics, resistome

## Abstract

Winter tourism can generate environmental pollution and affect microbial ecology in mountain ecosystems. This could stimulate the development of antibiotic resistance in snow and its dissemination through the atmosphere and through snow melting. Despite these potential impacts, the effect of winter tourism on the snow antibiotic resistome remains to be elucidated. In this study, snow samples subjected to different levels of anthropogenic activities and surrounding forest were obtained from the Sudety Mountains in Poland to evaluate the impact of winter tourism on snow bacteria using a metagenomic approach. Bacterial community composition was determined by the sequencing of the V3-V4 hypervariable region of the 16S rRNA gene and the composition of the antibiotic resistome was explored by metagenomic sequencing. Whereas environmental factors were the main drivers of bacterial community and antibiotic resistome composition in snow, winter tourism affected resistome composition in sites with similar environmental conditions. Several antibiotic resistance genes (ARGs) showed a higher abundance in sites subjected to human activities. This is the first study to show that anthropogenic activities may influence the antibiotic resistome in alpine snow. Our results highlight the need to survey antibiotic resistance development in anthropogenically polluted sites.

## Introduction

Mountains, and especially their snow cover, are sensitive indicators of climate change. Mountains support roughly one-third of all land-dwelling species and supply water for nearly half the global population (Körner and Paulsen, 2004). Mountains are warming at rates similar to the Arctic (Mountain Research Initiative EDW Working Group, 2015), which in turn is warming more than twice as fast as the global average (Screen and Simmonds, 2010). This has an impact on the environment (snow onset and duration, floods, changes in the distribution of species, and ecosystems) and society (summer and winter tourism). One major source of uncertainty is the extent to which human activities interact with climatic factors to modify biogeochemical processes, and ecosystem health and functioning. Among the activities known to affect alpine ecosystems, tourism (Ballantyne and Pickering, 2015; Mandaric et al., 2017; Barros et al., 2020) can have both positive and negative effects on mountain ecosystems, communities, and economies (United Nations Environment Programme, 2007). Tourism activities often involve the development and intense use of tracks, paths and sport slopes by vehicles, non-motorized transport, and pedestrian traffic. Visitor presence is also usually concentrated in small areas and contribute to increased noise and waste. The negative environmental effects of tourism can include devegetation, soil erosion, alteration of critical landscapes and water flows, water and air pollution, and wildlife relocation and behavioral changes (United Nations Environment Programme, 2007). The introduction of exotic and invasive species and diseases can also have a significant negative impact on the environment (Cowan et al., 2011).

The snow cover on mountains is connected to the atmosphere, which is considered to be one of the main sources of microorganisms in snow and is responsible for the transport of atmospheric dust and its inclusion in snow (Els et al., 2019, 2020; Maccario et al., 2019). Antibiotic resistance genes (ARGs) and pathogens contaminating the atmosphere can be deposited on Earth through snowfall and could increase their dissemination (Shen and Yao, 2013; Zhu et al., 2020). In addition, microorganisms present in snow are better adapted to atmospheric transport than other microorganisms (Harding et al., 2011), so antibiotic resistant bacteria (ARB) from contaminated snow could potentially be transported to remote environments. ARB and ARGs could also be transferred to freshwater through snow melting (Rogers et al., 2004), and thus increase the risk of waterborne disease.

The objective of this study was to evaluate the effect of winter tourism on snow-covered mountain microbial ecosystems. We hypothesized that environmental factors, such as surrounding forest, would be the main drivers of bacterial community and resistome composition in snow. On the other hand, the transfer of human microbiome bacteria to cryosphere environments due to anthropogenic activities should be limited since human bacteria are unlikely to adapt to a cold environment. However, we hypothesized that changes in the resistome at the antibiotic resistance gene level could be induced by human activities. To test this hypothesis, snow samples were obtained from two watersheds located in the Karkonosze National Park in the Sudety Mountains in Poland: an unaffected area and a catchment subjected to winter tourism. Samples with different levels of surrounding forest were recovered from both catchments. The effects of environmental and anthropogenic factors on the snow microbiome (bacterial community numbers and composition) and resistome (size and composition of the antibiotic resistome) were evaluated using 16S rRNA gene and metagenomic analyses, respectively.

## Materials and Methods

### Snow Sampling

Snow samples were obtained from two watersheds located in the Karkonosze National Park in the Sudety Mountains in Poland (Supplementary Figures S1, S2): an unaffected Czarny Kocioł Jagniątkowski catchment and a Kocioł Małego Stawu catchment with well documented human activities such as tourism and cottage development.

Samples with different levels of surrounding forest were recovered from both catchments. NP 1–10 samples from the unaffected Czarny Kociol Jagniatkowski catchment (low anthropogenic impact) were obtained from open spaces, whereas NP 11–20 were sampled in the forested area. S samples were obtained from paths in the Kociol Malego Stawu catchment with frequent human transit, SF were obtained from the surrounding forest areas, and LB were sampled from the ridge of the catchment in the open, treeless, less transited and windier areas. Vegetation levels in the different sites analyzed in this study were evaluated using the Normalized Difference Vegetation Index (NDVI; Supplementary Figure S3). Therefore, these samples cover a range of human activity and surrounding vegetation (Supplementary Figure S4).

### Snow Sample Processing

Samples were taken in March 2020 for microbial and chemical analyses and left at room temperature until melted. At this time of the year, human activity in the human-impacted catchment is estimated to be 100–1,000 times higher than that of the unaffected catchment. Three kilograms of snow were collected in sterile bags and transported to the field laboratory on a pulka using skis. They were melted at the field laboratory and filtered immediately on site. From the melted water, the microbial fraction of snow was recovered by filtering melted water through 0.2 μm Nucleopore membranes (Whatman, Maidstone, United Kingdom). Filters were then frozen at −20°C until DNA extraction. For chemical analyses, melted snow samples were filtered through 0.45-μm PES filters (Whatman, Maidstone, United Kingdom). The coordinates and elevation of each sample as well as the volume of sample that was filtered are shown in Supplementary Table S1.

### Physiochemical Analyses

One aliquot of filtered water was acidified with trace metal grade nitric acid to pH <2 and analyzed for trace element detection without further preparation. The other non-acidified aliquot was used to determine pH and specific conductivity by the microcomputer multifunction meter ELMETRON CX-551 (Supplementary Figure S5).

The inductively coupled plasma mass spectrometry system PlasmaQuant MS Q (AnalytikJena, Germany) was used to determine the concentration of 62 elements in snow. Standard conditions were used: radio frequency (RF) power 1.35 kW, plasma gas flow 9.0 l min^−1^, nebulizer gas flow 1.05 L min^−1^, auxiliary gas flow 1.5 L min^−1^, and sampling depth 5.0 mm. Signal was measured in five replicates (10 scans each). The isobaric interferences were reduced using the integrated Collision Reaction Cell (iCRC) working sequentially in three modes: without gasses addition, with helium as collision gas and hydrogen as reaction gas. Sc45, Y89, Rh103, and Ir193 were used as internal standards. Traceability was controlled using certified reference material EnviroMAT Drinking Water Low EP-L-4 (SCP Science, Canada) and the recovery (80%–120%) was acceptable for most of the determined elements. For not certified elements, the recovery in standard addition method was performed. Detection limits were determined in blank measurements, and trace elements for which more than 25% of the measurements were below the detection limit were excluded from further analyses (Supplementary Table S2). For the other elements, concentrations below detection limit were replaced by one-half of detection limit (Helsel and Hirsch, 1992, 2002). Element concentration in snow samples was used to calculate the enrichment factor (Supplementary Table S3) using the ratio of each element to barium in snow samples divided by the ratio of each element to barium in the upper continental crust (UCC; Rudnick and Gao, 2003), as in the following formula:


$$EF_{x}=\frac{\frac{X_{snow}}{Ba_{snow}}}{\frac{X_{UCC}}{Ba_{UCC}}}$$

where EF*_x_*, enrichment factor of element *x*; X_snow_/Ba_snow_, ratio of element *x* and Ba concentrations in melted snow samples; and X_UCC_/Ba_UCC_, ratio of element *x* and Ba in the UCC.

Barium has been used as a crustal tracer in other studies evaluating element concentrations in snow pack given its solubility, abundance, and the negligible effect of sea spray (Spolaor et al., 2021). UCC element concentrations were obtained from the article of Rudnick and Gao (2003). Kruskal–Wallis tests were used to compare the enrichment factors of each element between sites. The elements for which significant differences (significance defined here as *p* < 0.05) were found between sites are shown in Supplementary Table S4.

### DNA Extraction and 16S rRNA Gene Amplification by qPCR

Microbial DNA was extracted from 0.2-μm filters using the DNeasy PowerWater Kit (QIAGEN, Hilden, Germany) and eluted in 100 μl of Solution EB (QIAGEN). The size of the total bacterial community was estimated by quantifying the V3 region of the 16S rRNA gene by qPCR using the primers 341F (5′-CCT ACG GGA GGC AGC AG- 3′) and 534R (5′-ATT ACC GCG GCT GCT GGC A-3′; Muyzer et al., 1995; Watanabe et al., 2001). qPCR assays were carried out using the Corbett Rotor-Gene 6000 in a 20 μl reaction volume containing GoTaq qPCR Master Mix (Promega, Madison, Wisconsin, United States), 0.75 μM of each primer and 2 μl of DNA. Two non-template contamination controls were also included in all the assays. Standard curves were obtained using 10-fold serial dilutions of a linearized plasmid pGEM-T Easy Vector (10^2^–0^7^ copies) containing the 16S rRNA of *Pseudomonas aeruginosa* PAO1. Cycling conditions for qPCR amplification were 95°C for 2 min followed by 35 cycles of 95°C for 15 s, 60°C for 30 s and 72°C for 30 s. Melting curves were generated after amplification by increasing the temperature from 60°C to 95°C (5 s/°C). Melting peaks of 85.6°C ± 2°C were obtained from standards and snow DNA samples. The amplification threshold was of 0.3596 (normalized fluorescence, maximum 1). qPCR efficiency was equal to 1.04 and the R^2^ linearity coefficient was equal to 0.986. The limit of detection was 20 copies and the limit of quantification was 200 copies. The number of copies of the 16S rRNA gene were normalized per liter of melted snow.

### 16S rRNA Gene Sequencing and Analysis

The V3-V4 hypervariable regions of bacterial 16S rRNA gene were amplified from 3 μl of DNA using the Platinum Taq Polymerase (Invitrogen, Carlsbad, California, United States), forward S-D-Bact-0341-b-S-17 with Illumina adapter (5’-TCG TCG GCA GCG TCA GAT GTG TAT AAG AGA CAG TCG TCG GCA GCG TCA GAT GTG TAT AAG AGA CAG CCT ACG GGN GGC WGC AG-3′) and reverse S-D-Bact-0785-a-A-2 with Illumina adapter (5’-GTC TCG TGG GCT CGG AGA TGT GTA TAA GAG ACA GGT CTC GTG GGC TCG GAG ATG TGT ATA AGA GAC AGG ACT ACH VGG GTA TCT AAT CC-3′) primers (Klindworth et al., 2013). Amplification conditions were as follows: 95°C for 3 min followed by 35 cycles of 95°C for 30 s, 55°C for 30 s, and 72°C for 30 s, and a final extension step at 72°C for 5 min. DNA libraries were prepared from amplified products based on Illumina’s “16S Metagenomics Library Prep Guide” (15044223 Rev. B) using the Platinum Taq DNA Polymerase (Invitrogen) and the Nextera XT Index Kit V2 (Illumina, San Diego, California). DNA sequencing with a 15% PhiX spike-in was performed using the MiSeq System and the MiSeq Reagent Kit v2 (Illumina). Reads were trimmed to meet a quality score of Q20. Then, pair-ended reads were assembled using PANDAseq (Masella et al., 2012) at a sequence length between 410 and 500 bp and an overlap length between 20 and 100 bp, using the rdp_mle algorithm. Each of the DNA sequences was annotated to the genus level using the Ribosome Data Project (RDP) database and the RDP Bayesian classifier using an assignment confidence cut-off of 0.6 (Wang et al., 2007). Obtained sequencing depths are plotted in Supplementary Figure S6A. Contaminant sequences from blanks and non-template controls were removed using the decontam package in R (Davis et al., 2017) and genera that had less than 10 sequences annotated in the ensemble of samples were removed. Then, genus richness, evenness and diversity were calculated using the vegan package in R (Oksanen et al., 2019). In addition, genus relative abundances were calculated and the average abundance of the 24 most abundant genera was compared between sites. A NMDS analysis based on Bray-Curtis distances was performed using the vegan package in R.

### Metagenomic Sequencing and Analysis

DNA was quantified using the Qubit High Sensitivity dsDNA Kit (Invitrogen). Metagenomic libraries were prepared from <1 ng of DNA using the Nextera XT Library Pep Kit and Indexes (Illumina), as detailed in Illumina’s “Nextera XT DNA Library Prep Kit” reference guide (15,031,942 v03). DNA sequencing with a 1% PhiX spike-in was performed using the MiSeq System and the MiSeq Reagent Kit v2 (Illumina). Sequencing depths are plotted on Supplementary Figure S6B. Short reads were submitted to an ARG screening. Firstly, reads were trimmed using the Fastq Quality Trimmer tool of the FASTX-Toolkit. Nucleotides that did not meet a minimum quality score of Q20 were trimmed from the sequences, and sequences shorter than 100 nucleotides after trimming were removed. Then, reads from R1 and R2 were concatenated and blasted against the CARD antibiotic gene database (Alcock et al., 2020) using Diamond (Buchfink et al., 2015). The obtained results were filtered at a minimum identity of 60%, a minimum length of 33 amino acids and an e-value of 10e^−5^, and the best hit was chosen. Singletons and ARGs present in blanks were removed. Then, ARG copies were normalized per liter of melted snow or per number of copies of the 16S rRNA gene. Both ARG reads normalized by liter of melted snow and normalized by 16S rRNA gene copies were grouped by antibiotic class and their average abundance was compared between sites. In addition, a NMDS analysis was performed on ARG abundances per liter of snow using the vegan package in R. Finally, statistical differences in ARG abundance between sites were found using the DESeq2 package in R (Love et al., 2014). Log2FoldChange values were adjusted using the Approximate Posterior Estimation for generalized linear model or apeglm. Results with a log2FoldChange higher than ±2 and an adjusted value of *p* lower than 0.001 were plotted using the ggplot2 package in R.

### Statistical Analyses

Statistical analyses were performed for all the numerical parameters evaluated in this study: NDVI, pH, conductivity, concentration of trace elements, sequencing depth, 16S rRNA gene copies per L of melted snow, bacterial richness, evenness and diversity, ARG copies per L of melted snow, ARG copies per 16S rRNA gene copies. Data normality was checked for each parameter using the Shapiro–Wilk test. Statistical differences between sites were evaluated using ANOVA tests and pairwise *t*-student tests for data that showed a normal distribution and Kruskal–Wallis and pairwise Wilcoxon signed-rank tests for data that showed a non-normal distribution. All statistical analyses were done using the ggpubr package in R (Kassambara, 2020).

In addition, statistical differences in genus and ARG abundance between sites were found using the DESeq2 package in R (Love et al., 2014). Log2FoldChange values were adjusted using the Approximate Posterior Estimation for generalized linear model or apeglm. Results with a log2FoldChange higher than ±2 and an adjusted value of *p* lower than 0.05 for genus abundance and 0.001 for ARG abundance were plotted using the ggplot2 package in R.

### Impact of Physiochemical Parameters on Bacterial Community and Resistome Composition

In order to determine whether physiochemical factors have a significant impact on bacterial community and resistome composition, environmental factors (NDVI, pH, conductivity, and trace elements that showed EF values higher than 5 and significant differences between sites) were fit onto the NMDS ordinations based on genus or ARG abundance using the envfit function in R with 999 permutations. The significance of the impact of each parameter on bacterial and resistome composition was calculated (Supplementary Tables S5, S6) and the projection of environmental vectors onto sample points was added to the NMDS analyses. In addition, sites were divided by surrounding vegetation (low for LB and NP 1–10, high for NP 11–20, S and SF) and human impact (low for NP 1–20 and high for S and SF – LB was excluded from the analysis because it represented extreme environment conditions). PERMANOVA analyses were done on the NMDS ordinations using the vegan package in R to determine whether vegetation or human activity had a significant impact on the composition of bacterial communities and antibiotic resistome in snow.

## Results

### Physiochemical Characterization of Snow

No significant differences in pH were found between sites (Supplementary Figure S5A). On the other hand, the conductivity in LB (the site with the most scarce vegetation and harsher environmental conditions) was significantly lower than in any other site. NP 1–10 (open spaces from the unaffected catchment) showed a significantly higher conductivity than S (paths from the human-impacted catchment; Supplementary Figure S5B). In addition, only a few elements had an enrichment factor (EF) higher than 5 (Ni, Cu, Zn, Na, B, Mo, Pt, Pb, As, and Cd), and from these only Mn, Ni, Cu, Cd, and B had significant differences between site (Table 1). Overall, similar EF of trace elements were found between NP 1–10, NP 11–20 (forested areas from the unaffected catchment), S and SF (forested areas from the human-impacted catchment), which are subjected to different levels of anthropogenic presence (Supplementary Table S3). Cadmium (Cd) was an exception as it had significantly higher EF levels at NP 1–10 than at S (Supplementary Table S4). However, some trace elements had significant differences between LB and other sites (Supplementary Table S4). These were overall lower at LB (Ce, V, K, Mn, B, Rb, Zr, and La), except for Copper (Cu) and Nickel (Ni), which were higher at LB than at other sites.

**Table 1:** Enrichment factors (EFs) for trace elements that showed EF values higher than 5 and significant differences between sites.

Element	Site	Average	SD	Significant differences
Mn	LB (*n* = 10)	0.69	0.34	LB < NP 11–20, S, SF
	NP 1–10 (*n* = 10)	1.28	0.46	
	NP 11–20 (*n* = 10)	1.91	1.03	
	S (*n* = 10)	2.46	1.83	
	SF(*n* = 9)	4.71	3.15	
Ni	LB (*n* = 10)	23.43	9.18	LB > NP 1–10, NP 11–20, S
	NP 1–10 (*n* = 10)	11.09	4.79	
	NP 11–20 (*n* = 10)	11	6.15	
	S (*n* = 10)	10.15	4.46	
	SF(*n* = 9)	13.68	5.57	
Cu	LB (*n* = 10)	79.09	93.39	LB > NP 1–10, S
	NP 1–10 (*n* = 10)	30.06	56.01	
	NP 11–20 (*n* = 10)	28.52	30.13	
	S (*n* = 10)	13	10.6	
	SF(*n* = 9)	28.74	17.67	
Cd	LB (*n* = 10)	222.58	102.39	NP 1–10 > S
	NP 1–10 (*n* = 10)	371.34	170.78	
	NP 11–20 (*n* = 10)	316.84	168.88	
	S (*n* = 10)	196.06	75.75	
	SF(*n* = 9)	215.05	110.5	
B	LB (*n* = 10)	38.56	17.63	LB < NP 1–10
	NP 1–10 (*n* = 10)	97.99	54.15	
	NP 11–20 (*n* = 10)	50.1	25.63	
	S (*n* = 10)	49.15	21.33	
	SF(*n* = 9)	77.18	38.83	

LB: ridge from the catchment with human transit. NP 1–10: open spaces from the unaffected catchment. NP 11–20: forested area from the unaffected catchment. S: paths from the catchment with human transit. SF: forest areas from the catchment with human transit. Values of *p* are indicated in Supplementary Table S4.

**Figure 1:**
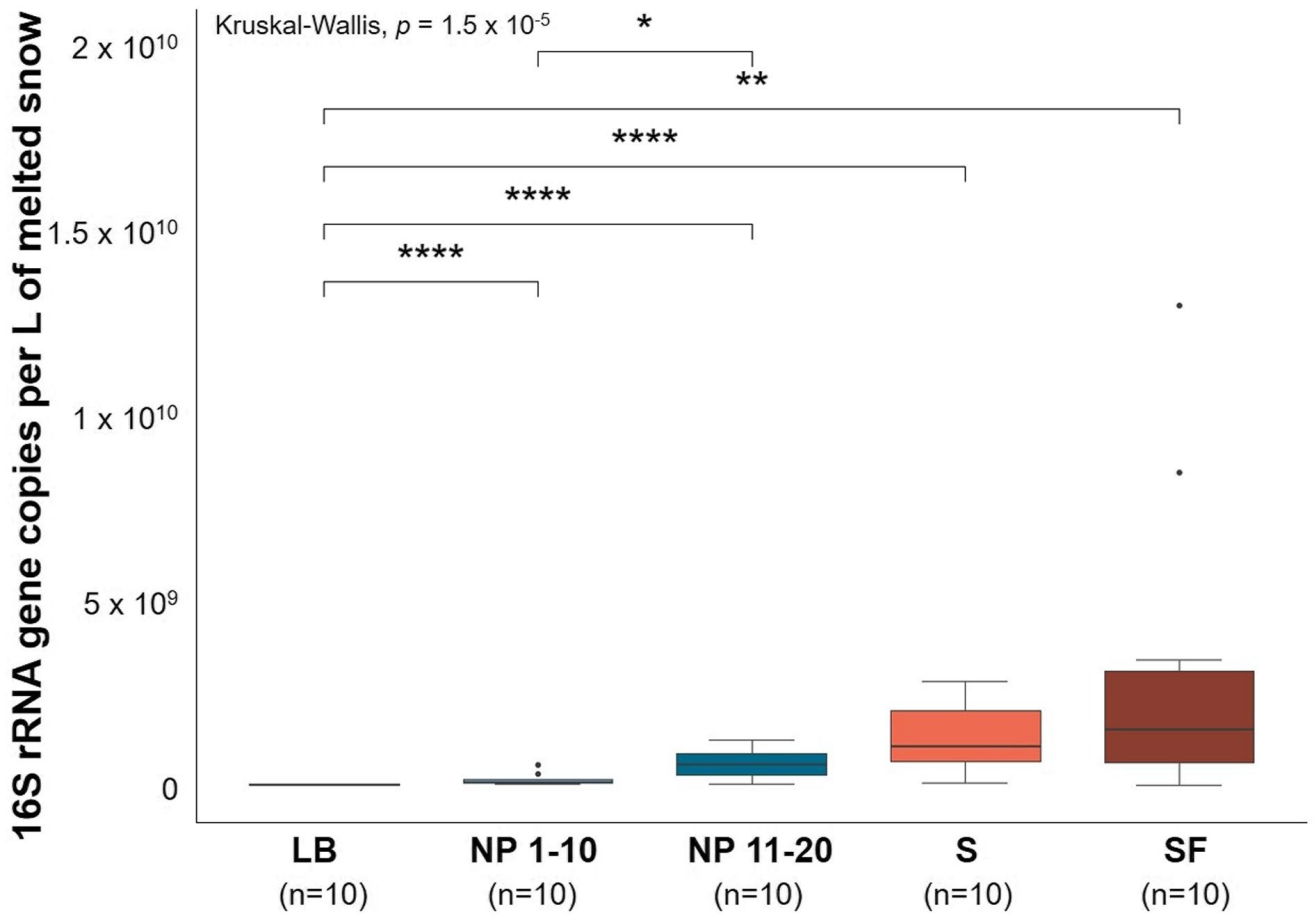
Estimates of bacterial biomass based on qPCR of the 16S rRNA gene from all sites. LB: ridge from the catchment with human transit. NP 1–10: open spaces from the unaffected catchment. NP 11–20: forested area from the unaffected catchment. S: paths from the catchment with human transit. SF: forest areas from the catchment with human transit. Copies were normalized per L of melted snow. qPCR efficiency = 1.04. *R*^2^ linearity coefficient = 0.986. Data normality was checked using the Shapiro–Wilk test (*p* = 5.03 × 10^−12^). Significant differences between sites were determined by pairwise Wilcoxon signed-rank tests. *p ≤ 0.05, **p ≤ 0.01, and ****p ≤ 0.0001. *n* = 10.

Samples that had significantly less surrounding forest (Supplementary Figure S3) also showed significantly lower microbial biomass than those in forested areas (Figure 1). LB showed significantly lower levels of vegetation and bacterial biomass than any other site. This was also observed at NP 1–10 when compared to NP 11–20. On the other hand, no significant difference in terms of bacterial biomass was found between sites that had different levels of anthropogenic activity but non-significant differences in surrounding forest.

### Effect of Environmental and Anthropogenic Factors on the Snow Microbiome

Environmental and anthropogenic factors did not have an impact on the genus richness observed in snow (Supplementary Figure S7A). Conversely, the genus evenness and diversity measured in LB, the site subjected to the most extreme conditions, were significantly higher than those at any other site (Supplementary Figures S7B,C).

**Figure 2:**
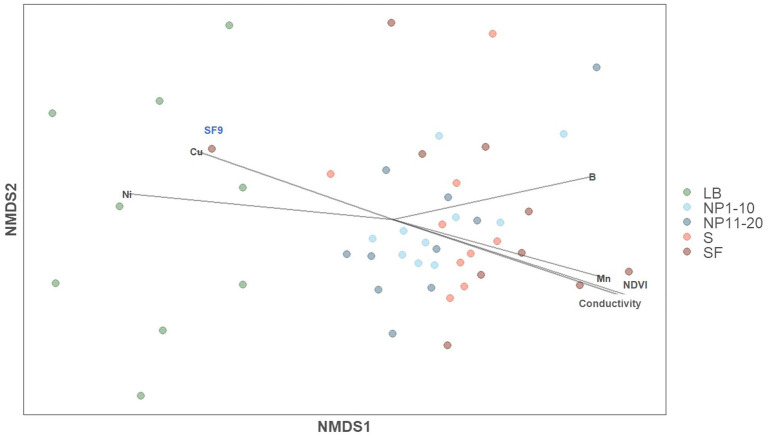
Impact of physiochemical factors on bacterial community composition (NMDS analysis based on Bray-Curtis distances). NMDS stress = 0.132. LB: ridge from the catchment with human transit. NP 1–10: open spaces from the unaffected catchment. NP 11–20: forested area from the unaffected catchment. S: paths from the catchment with human transit. SF: forest areas from the catchment with human transit.

A non-metric multidimensional scaling (NMDS) analysis was applied to all the samples to determine whether surrounding forest and/or anthropogenic activity shaped bacterial community composition (Figure 2). Most of the samples from NP 1–10, NP 11–20, S and SF formed a cluster, whereas all LB samples clustered separately from the rest. The only sample from SF that was included in the LB cluster was SF9, which is geographically closer to LB than to the rest of samples in SF (Supplementary Figure S2). NDVI, conductivity and the concentration of Mn, Ni, Cu, and B showed significant correlations with bacterial community composition (Supplementary Table S5): Cu and Ni pointed to the LB cluster and the other factors to the cluster with the rest of the samples (Figure 2). PERMANOVA analyses showed that vegetation had a significant impact on overall bacterial community composition [Pr (>F) = 0.039], whereas human activity did not [Pr (>F) = 0.091].

*Granulicella* was the predominant genus in all sites and *Diplororickettsia* was more abundant in SF than in any other site (Supplementary Figure S8). Overall, the most abundant genera had similar relative abundances between sites except for LB. Consistently, whereas several genera were more abundant in NP 11–20, S, and SF than in LB (Supplementary Figures S9B-D), few or no statistical differences in genus abundance were found between any other site (Supplementary Figures S9A, E-H).

### Effect of Environmental and Anthropogenic Factors on the Snow Antibiotic Resistome

**Figure 3:**
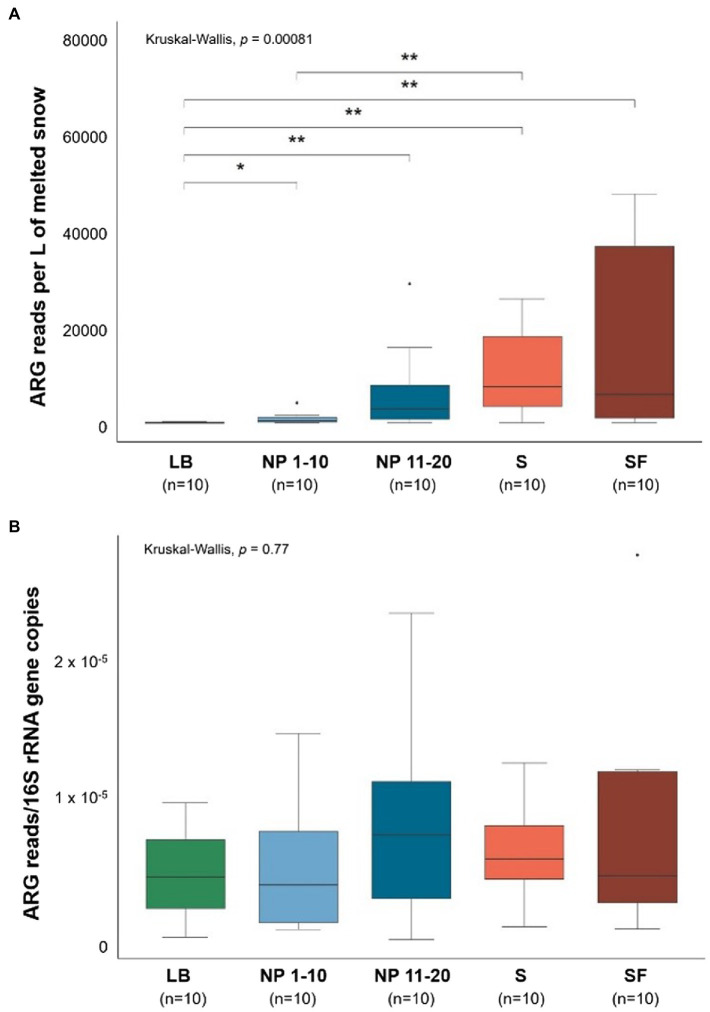
Size of the snow antibiotic resistome. LB: ridge from the catchment with human transit. NP 1–10: open spaces from the unaffected catchment. NP 11–20: forested area from the unaffected catchment. S: paths from the catchment with human transit. SF: forest areas from the catchment with human transit. Total ARG copies per site normalized by **(A)** L of melted snow and **(B)** by copies of the 16S rRNA gene. Data normality was checked using the Shapiro–Wilk test (**A**: *p* = 1 × 10^−9^ and **B**: *p* = 7.83 × 10^−5^). Significant differences between sites were determined by pairwise Wilcoxon signed-rank tests. *p ≤ 0.05 and **p ≤ 0.01. *n* = 10.

The number of ARG reads obtained from the metagenomic sequencing of snow DNA was normalized per liter of melted snow (Figure 3A) and per number of copies of the 16S rRNA gene (Figure 3B).

Significantly less ARG reads per L of melted snow were obtained from the sequencing of LB samples than from any other site. In addition, NP 1–10 had fewer ARG sequences per L of melted snow than S. On the other hand, when the size of the antibiotic resistome was normalized by bacterial biomass (i.e., by number of copies of the 16S rRNA gene), no significant differences were found between sites.

**Figure 4:**
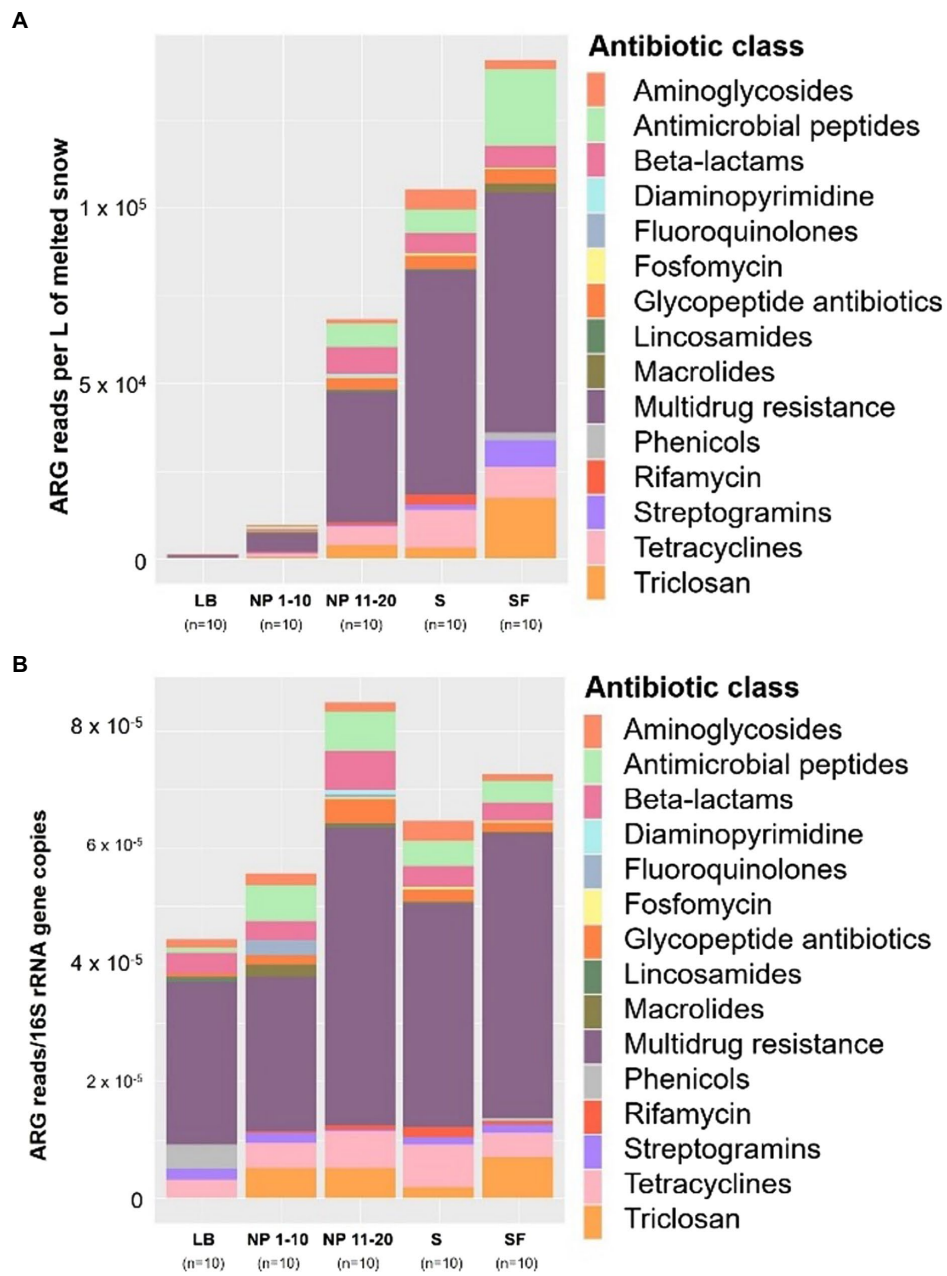
Total ARG copies per site normalized **(A)** by L of melted snow and **(B)** by copies of the 16S rRNA gene (right) grouped by antibiotic class. LB: ridge from the catchment with human transit. NP 1–10: open spaces from the unaffected catchment. NP 11–20: forested area from the unaffected catchment. S: paths from the catchment with human transit. SF: forest areas from the catchment with human transit. *n* = 10.

The composition of the antibiotic resistome from the different sites was compared. When normalized by L of melted snow (Figure 4A), sites with less surrounding forest (i.e., NP 1–10 and LB) had a lower abundance of genes belonging to all the detected antibiotic classes than at any other site. In addition, S and SF had higher numbers of multidrug resistance genes than NP 11–20. An increase in ARGs conferring resistance to antimicrobial peptides, streptogramins and triclosan was also observed at SF, whereas S showed higher levels of rifamycin and tetracycline resistance genes than any other site. On the other hand, all these differences, except for the higher presence of rifamycin resistance genes in S, are reduced when normalizing by bacterial biomass (Figure 4B). In addition, higher levels of phenicol and fluoroquinolone resistance genes per bacterial community size are observed in LB and in NP 1–10, respectively. Finally, a NMDS analysis showed that overall samples showed relatively low dissimilarity except for some LB samples and NP12 (Supplementary Figure S10). Physiochemical factors did not show any significant impact on the composition of the bacterial resistome. Vegetation showed a significant impact on overall resistome community composition according to the PERMANOVA test [Pr (>F) = 0.001], whereas no significant impact was found for human activity [Pr (>F) = 0.073].

**Figure 5:**
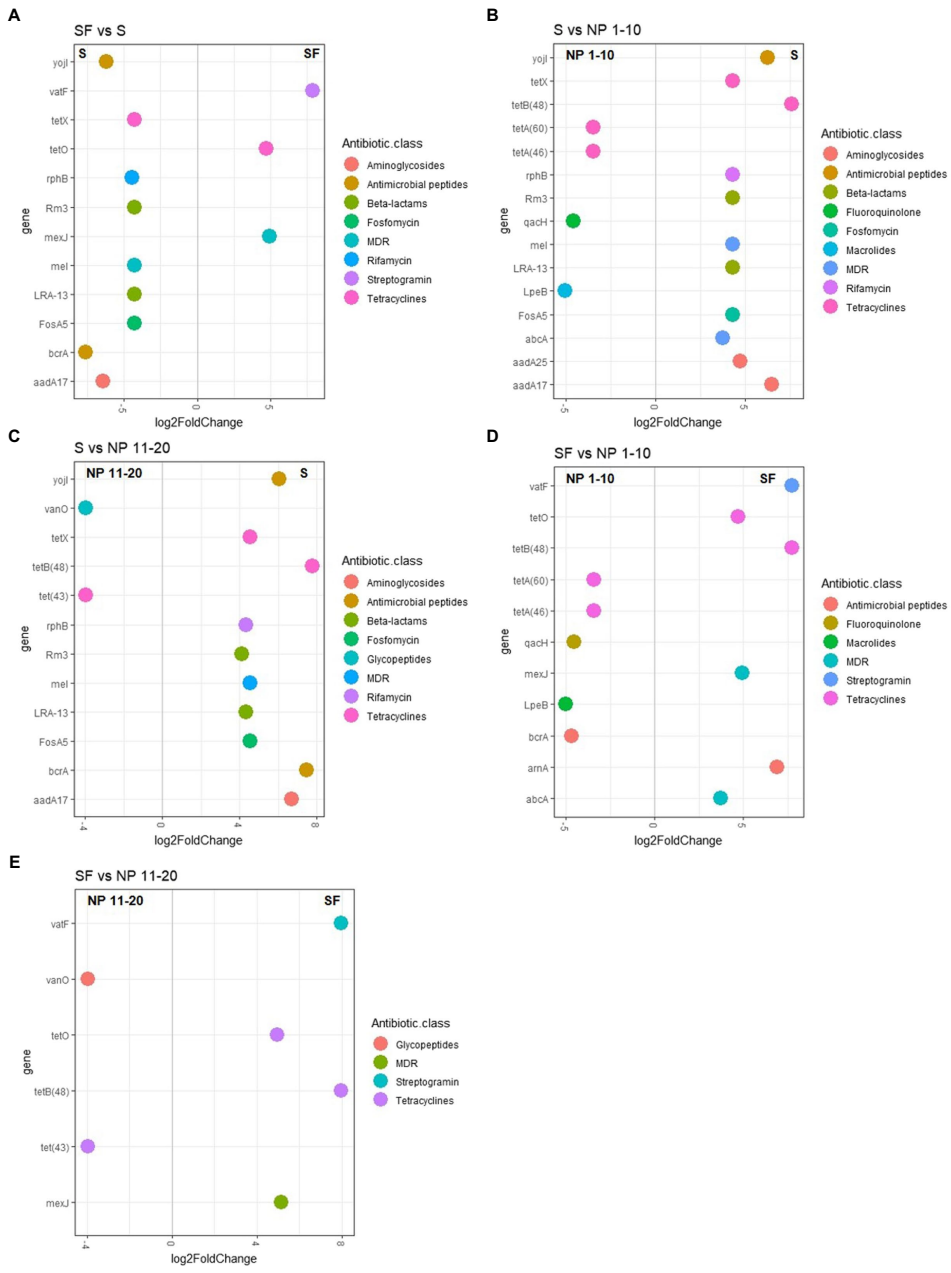
ARG abundance pairwise comparisons between sites that showed no significant difference in vegetation levels. **(A)** SF versus S; **(B)** S versus NP 1–10; **(C)** S versus NP 11–20; **(D)** SF versus NP 1–10; **(E)** SF versus NP 11–20. NP 1–10: open spaces from the unaffected catchment. NP 11–20: forested area from the unaffected catchment. S: paths from the catchment with human transit. SF: forest areas from the catchment with human transit. Only results with a log2FoldChange ± 2 and an adjusted value of *p* lower than 0.001 are shown. *n* = 10.

All sites had several ARGs in a higher abundance than LB (Supplementary Figure S11). Since the sites with higher levels of surrounding forest showed a higher ARG abundance, ARG abundance differences were evaluated between sites that showed no significant differences in terms of vegetation (Supplementary Figure S3) but were submitted to different levels of human presence (Figure 5). Several genes showed a higher abundance in S (samples from the paths with the highest human presence) compared to SF (Figure 5A), NP 1–10 (Figure 5B) and NP 11–20 (Figure 5C). These genes were *aadA17,* involved in aminoglycoside resistance, the tetracycline resistance gene *tetX*, the confer resistance to the rifamycin resistance gene *rphB*, the beta-lactam resistance genes *rm3* and *LRA-13*, the multidrug resistance gene *meI* and the Fosfomycin resistance gene *fosA5*. Although some differences were found when SF was compared to NP 1–10 (Figure 5D) and NP 11–20 (Figure 5E), the composition of the resistome was more similar between these sites than between any of them and S. Similarly, whereas some ARGs were more abundant in SF, NP 1–10, and NP 11–20 than in S, more genes showed a higher abundance in S than in any of these sites (Figures 5A–C).

## Discussion

The main goal of this study was to determine whether human presence leaves a detectable trace in the snow microbiome and its associated resistome as hypothesized. Although the differences observed between sites mostly reflected differences in the presence or absence of a surrounding forest, our results suggest that human presence could have an impact on the composition of the antibiotic resistome in snow.

Overall, inorganic composition of snow was similar between sites subjected to different levels of anthropogenic activities except for LB, which was subjected to more extreme environmental conditions. Most of the elements measured showed enrichment factor values lower than 5, which indicates a lack of metal pollution caused by human activity in snow cover (Barbante et al., 2017; Spolaor et al., 2021). These elements are probably of natural origin and likely to be related to deposition of eolian-derived material. In addition, the overall similar inorganic composition found in sites subjected to winter tourism (S and SF) and unaffected sites (NP 1–10 and NP 11–20) suggests that tourism activity does not affect trace element concentration in the snowpack. Since no major differences were found in the chemical composition of sites with similar levels of vegetation, some of the differences observed at the resistome level between sites could be attributable to winter tourism instead of environmental variables. However, some chemical parameters that were not measured in this study, such as organic carbon concentrations, could also impact the composition of the snow resistome. On the other hand, our findings confirm previous results suggesting that some of these elements (Ni, Cu, and Zn), which are Potentially Harmful Trace Elements (Le Roux et al., 2016), are observed in mountain peatlands in the West Sudetes (Fiałkiewicz-Kozieł et al., 2020). The higher EF of copper and nickel in LB than in the other sites could be related to long-range transport of these industrial-derived pollutants from western directions, as suggested by Fiałkiewicz-Kozieł et al. (2020).

The abundance and composition of bacterial communities were mainly driven by environmental factors and overall reflected differences in the levels of surrounding forest between sites. Samples that had the lowest biomass and the more distinct bacterial composition (LB) come from the site that has the higher elevation and is subjected to the most extreme environmental conditions (wind and cold) and scarcest forest. In addition, only the extreme conditions and the virtually complete absence of trees associated with LB samples seem to have an effect on bacterial evenness and diversity (Supplementary Figure S7). The presence of trees was inversely related to a higher evenness and diversity of bacteria. Snowpack microbial communities are seeded from the atmosphere through wet and dry deposition processes (Maccario et al., 2014, 2019). Since the snow is coupled to the atmosphere, factors that affect airborne microbial communities, such as surrounding ecosystems and local meteorological conditions, could change the snow microbial community structure (Tignat-Perrier et al., 2019; Els et al., 2020). At sites where trees are scarce, microbial life would be expected to colonize snow mainly from the atmosphere and dust and lead to a more even community (Chuvochina et al., 2011; Courville et al., 2020). At sites with surrounding forest, the presence of trees might influence bacterial communities in two ways: (a) the phyllosphere community found on needles of trees could directly colonize the snow (Lindow and Brandl, 2003; Rúa et al., 2016) and (b) tree litter could be a source of organic matter that would indirectly influence the growth of snow microorganisms (Mahajan et al., 2016; Chae et al., 2019). Overall, vegetation seems to support a more abundant bacterial community as suggested by previous studies (Li et al., 2018; Chen et al., 2019). On the other hand, no remarkable effect of human presence on bacterial community structure was observed. Since human-microbiome bacteria are unlikely to proliferate and be active in extreme environments such as snow, direct biological contamination of anthropogenically-impacted snow should not have a strong effect on the snow microbiome. Therefore, a major impact of human presence on the size or the composition of the bacterial communities is unlikely. Furthermore, both sites subjected to human presence (S and SF) were forested areas that provide favorable conditions for bacteria. Thus, the potential added effects of human presence or any anthropogenic activity would not necessarily affect the core bacterial community.

In addition, the more favorable conditions for bacteria observed at sites with higher levels of surrounding forest are associated with a more abundant antibiotic resistome. All forested areas showed higher levels of antibiotic resistance at the genetic level, which was likely a consequence of the higher biomass. The hypothesis that ARG load was determined by bacterial abundance rather than by selective pressure was supported by the reduction of the differences between sites when the abundance of the antibiotic resistome was normalized by bacterial biomass. Although resistome composition was mainly influenced by environmental factors (i.e., surrounding forest) and no significant effect of human activity on overall resistome composition was observed, some effects attributed to anthropogenic impact were measured in S (Figure 5). This increased abundance of antibiotic resistance genes in S compared to sites that had similar levels of surrounding vegetation and lower human presence (NP 1–10, NP 11–20, and SF) was detected both at the antibiotic class and at the ARG level. Although this impact is fairly low, considering the low likelihood of adaptation of human bacteria to cold, extreme environments, the impact of humans on the snow microbiome was not expected to be the major driver. However, some factors related to human presence, such as an increased nutrient availability, could translate to changes at the resistome level. For example, a previous study has shown that anthropogenic waste can induce ARG spread without influencing community composition, and linked these changes to a higher macronutrient load in sewage (Lehmann et al., 2016). Based on our study, we were not able to determine the mechanisms underlying the increased abundance of ARGs in human-impacted snow, but our results raise concerns about the effect that winter tourism could have on antibiotic resistance development in the snow environment.

In conclusion, environmental (i.e., surrounding forest) and anthropogenic factors induced some changes in the snow microbiome and its associated resistome; and some micro-organisms responded to environmental changes in alpine snow. Although several studies have illustrated the impact of anthropogenic activity on the environmental resistome (Chen et al., 2013; Czekalski et al., 2015; Suzuki et al., 2015), this is the first study to observe that winter tourism could induce changes in the snow resistome without affecting the core bacterial community structure. Thus, anthropogenic activity could be an indirect source of environmental pollution and stimulate the development of antibiotic resistance in the snow microbiome that might be subsequently disseminated through the atmosphere and snow melting. This study provides insights into the potential impact of environmental and anthropogenic factors on snow microbial ecology and highlights the need for survey of antibiotic resistance development in sites affected by anthropogenic activities and the consequences that environmental pollution may have on antibiotic resistance dispersion.

## Data Availability Statement

The datasets presented in this study can be found in online repositories. The names of the repository/repositories and accession number(s) can be found at: https://www.mmnt.net/db/0/0/ftp.ec-lyon.fr/pub/ADN/, Sanchez-Cid_2022_snow_tourism.

## Author Contributions

CS-C, CK, KT, LS, BL, AN, and CL contributed to the study design and sampling. DK, BL, and AN designed the maps. DK calculated vegetation indexes. CS-C, CK, and CL extracted and sequenced DNA. PN analyzed trace elements in snow and conducted QC and QA. LS measured pH and conductivity and calculated enrichment factors. CS-C performed bioinformatic and statistical analyses. CS-C and CL wrote the manuscript. All authors participated in result discussion/interpretation and manuscript reviewing. All authors contributed to the article and approved the submitted version.

## Funding

This work was partially funded by the NAWA PHC Polonium 2020 program of the French-Polish Hubert Curien partnership [project number PPN/BFR/2019/1/00052/U/00001 (PL) and 45023QL (FR) in 2020]. Partial funding was received from the ANRT (Association Nationale de la Recherche et de la Technologie) and Promega Corporation, as part of the CIFRE PhD grant 2017/0486. BL and AN were also supported by the Institute of Geophysics, Polish Academy of Sciences within statutory activities no. 3841/E-41/S/2020 of the Ministry of Science and Higher Education of Poland.

## Conflict of Interest

Author CS-C was employed by company Promega France.

The remaining authors declare that the research was conducted in the absence of any commercial or financial relationships that could be construed as a potential conflict of interest.

## Publisher’s Note

All claims expressed in this article are solely those of the authors and do not necessarily represent those of their affiliated organizations, or those of the publisher, the editors and the reviewers. Any product that may be evaluated in this article, or claim that may be made by its manufacturer, is not guaranteed or endorsed by the publisher.
