# Supplementary material for: Environmental and Anthropogenic Factors Shape the Snow Microbiome and Antibiotic Resistome
Source: Front Microbiol. 2022 Jun 16;13:918622. doi: 10.3389/fmicb.2022.918622 (PMC9245712; doi:10.3389/fmicb.2022.918622)
Supplement: Supplementary file 1 [file Data_Sheet_1.PDF]

Supplementary Material

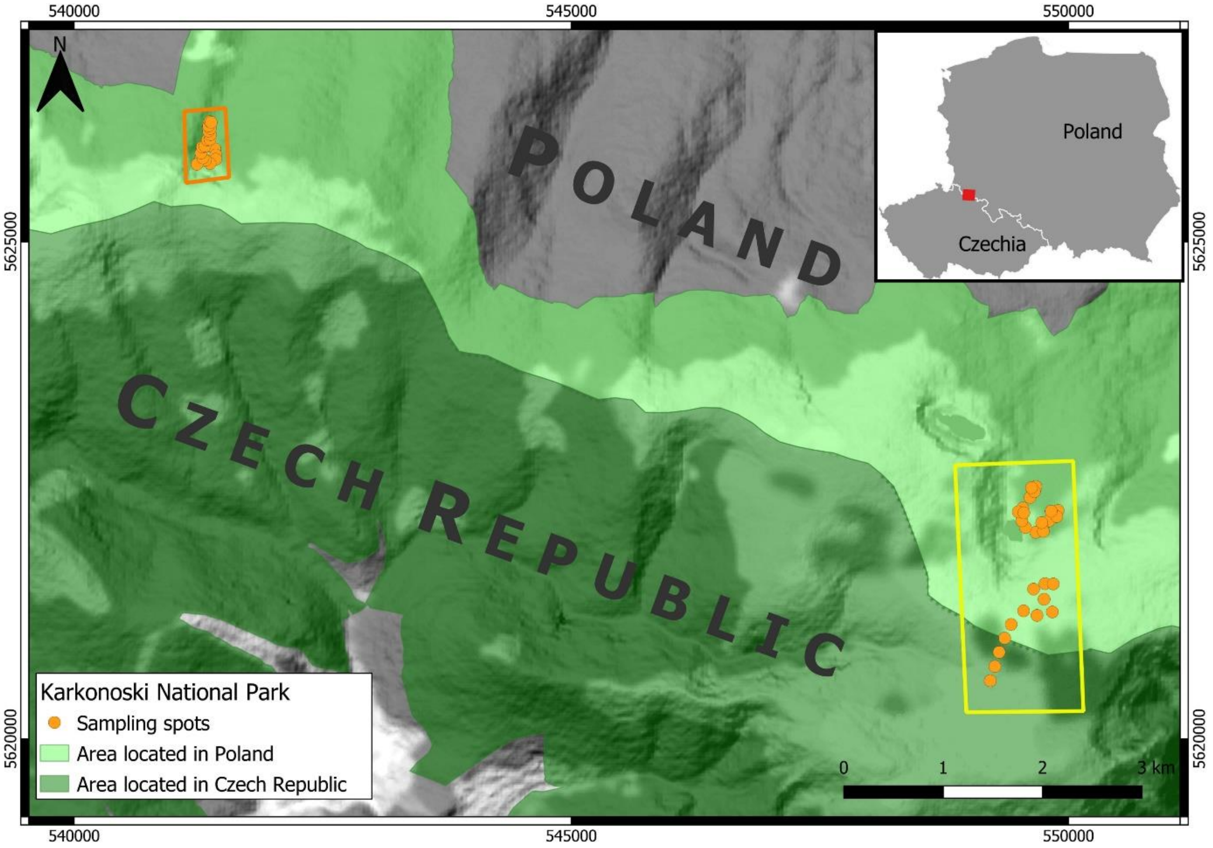

Figure S1. Geographical location of sampling sites. Czarny Kociol Jagniatkowski catchment (orange box) and the Kociol Malego Stawu catchment (yellow box).

A

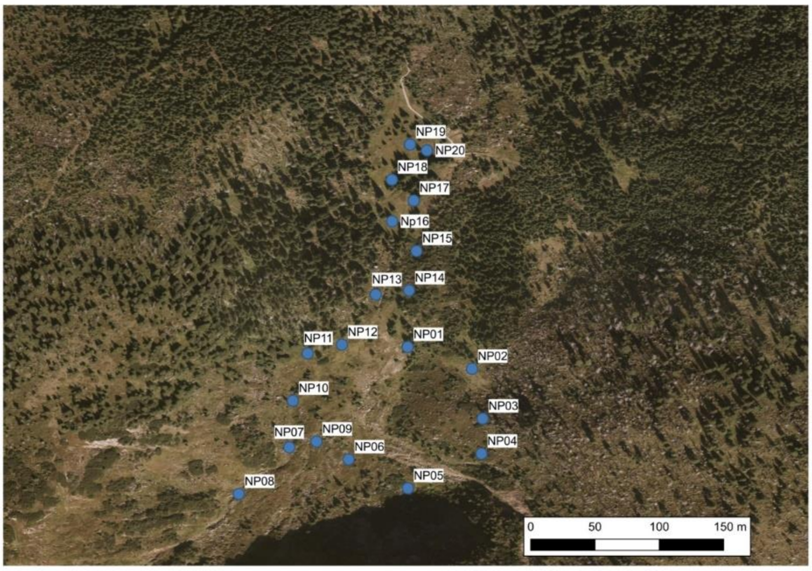

B

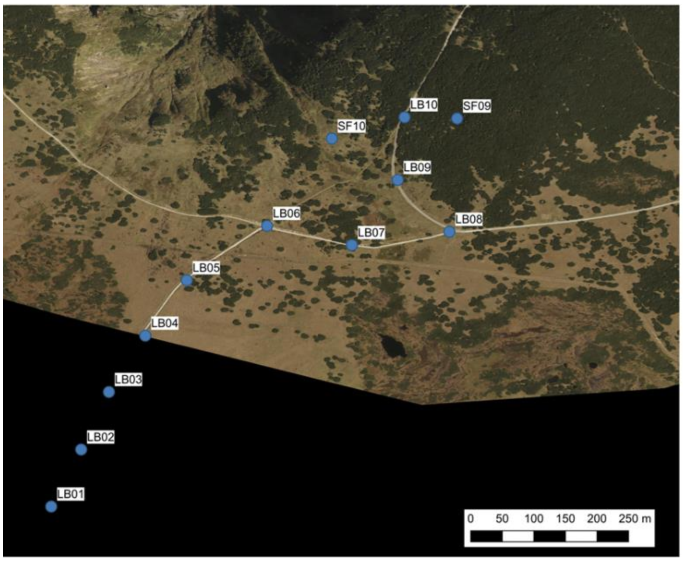

C

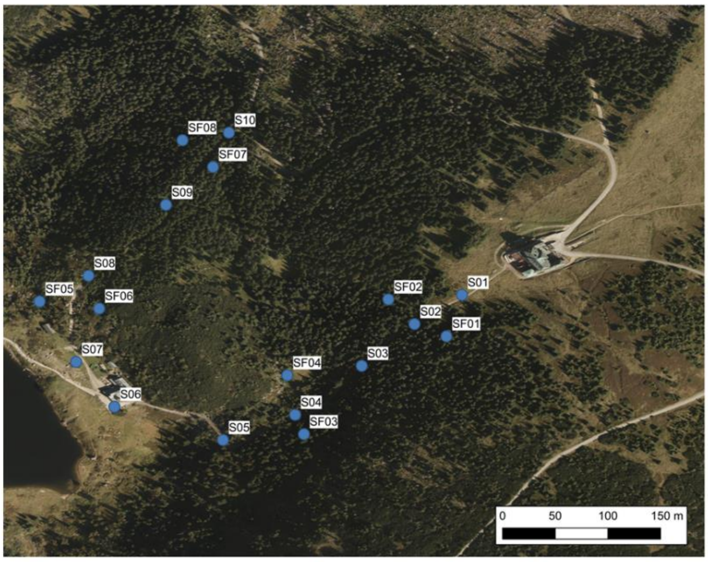

Figure S2. Map of the samples from the Czarny Kociol Jagniatkowski catchment (A) and the Kociol Malego Stawu catchment (B, C). LB1 to 3 were outside Polish territory in the Czech Republic and satellite images are not shown. Coordinate Reference System: WGS84.

Sample	Latitude	Longitude	Elevation (m)	Filtered volume (ml)	Sample	Latitude	Longitude	Elevation (m)	Filtered volume (ml)
**NP01**	50.78	15.59	1177	2000	**S06**	50.75	15.70	1190	1500
**NP02**	50.78	15.59	1161	2000	**S07**	50.75	15.70	1195	1500
**NP03**	50.78	15.59	1181	1500	**S08**	50.75	15.70	1202	1500
**NP04**	50.78	15.59	1187	2000	**S09**	50.75	15.70	1198	1400
**NP05**	50.78	15.59	1168	2000	**S10**	50.75	15.70	1209	1350
**NP06**	50.78	15.59	1173	2000	**SF01**	50.75	15.71	1290	500
**NP07**	50.78	15.59	1185	2000	**SF02**	50.75	15.71	1247	900
**NP08**	50.78	15.58	1209	2000	**SF03**	50.75	15.71	1250	500
**NP09**	50.78	15.59	1164	2000	**SF04**	50.75	15.70	1233	1500
**NP10**	50.78	15.59	1146	2000	**SF05**	50.75	15.70	1184	1500
**NP11**	50.78	15.59	1131	1500	**SF06**	50.75	15.70	1216	1500
**NP12**	50.78	15.59	817	2000	**SF07**	50.75	15.70	1184	500
**NP13**	50.78	15.59	812	2000	**SF08**	50.75	15.70	1179	500
**NP14**	50.78	15.59	811	2000	**SF09**	50.74	15.71	1410	1500
**NP15**	50.78	15.59	838	2000	**SF10**	50.74	15.70	1386	1500
**NP16**	50.79	15.59	807	2000	**LB01**	50.73	15.70	1413	1500
**NP17**	50.79	15.59	1092	2000	**LB02**	50.74	15.70	1416	1500
**NP18**	50.79	15.59	1097	2000	**LB03**	50.74	15.70	1424	1500
**NP19**	50.79	15.59	1093	1900	**LB04**	50.74	15.70	1424	1500
**NP20**	50.79	15.59	1095	1500	**LB05**	50.74	15.70	1428	1500
**S01**	50.75	15.71	1253	1500	**LB06**	50.74	15.70	1416	1500
**S02**	50.75	15.71	1248	1500	**LB07**	50.74	15.70	1425	1500
**S03**	50.75	15.71	1239	1500	**LB08**	50.74	15.71	1430	1500
**S04**	50.75	15.71	1233	1500	**LB09**	50.74	15.71	1410	1500
**S05**	50.75	15.70	1215	1500	**LB10**	50.74	15.71	1395	1500

Table S1. Latitude, longitude, elevation and filtered volume of each sample included in this study. Coordinate Reference System: WGS84.

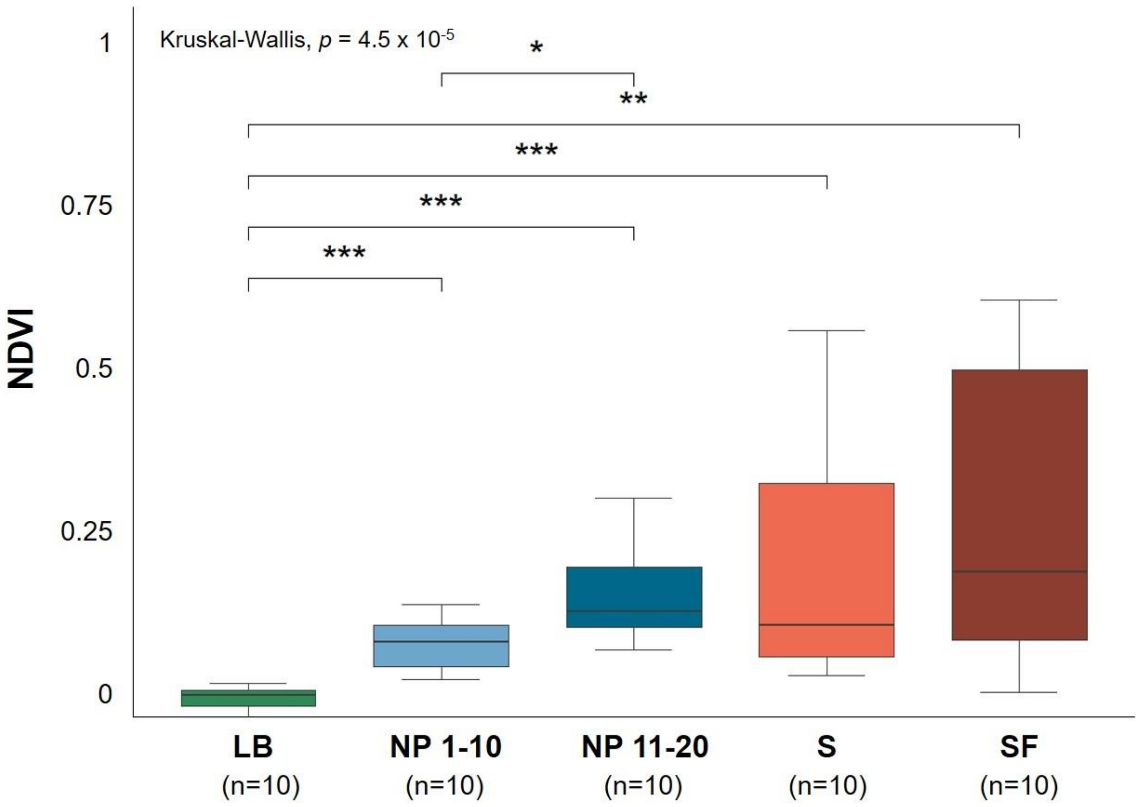

Figure S3. Surrounding vegetation per site. NP 1-10: open spaces from the unaffected catchment. NP 11-20: forested area from the unaffected catchment. S: paths from the catchment with human transit. SF: forest areas from the catchment with human transit. Vegetation levels were calculated using the Normalized Difference Vegetation Index (NDVI). Data normality was checked using the Shapiro-Wilk test (p = 1.39 x 10^{-6}). Significant differences between sites were determined by pairwise Wilcoxon signed-tank tests. *p-value ≤ 0.05. **p-value ≤ 0.01. *** p-value ≤ 0.001. n=10.

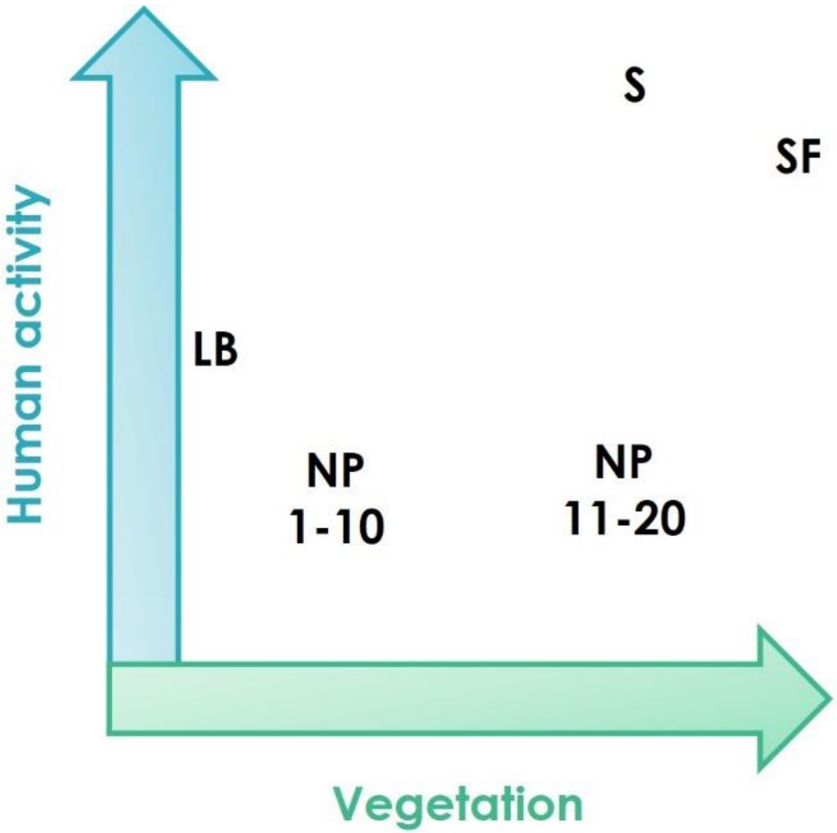

Figure S4. Diagram illustrating the gradient of human activity and surrounding vegetation that characterized the five areas analyzed in this study. NP 1-10: open spaces from the unaffected catchment. NP 11-20: forested area from the unaffected catchment. S: paths from the catchment with human transit. SF: forest areas from the catchment with human transit.

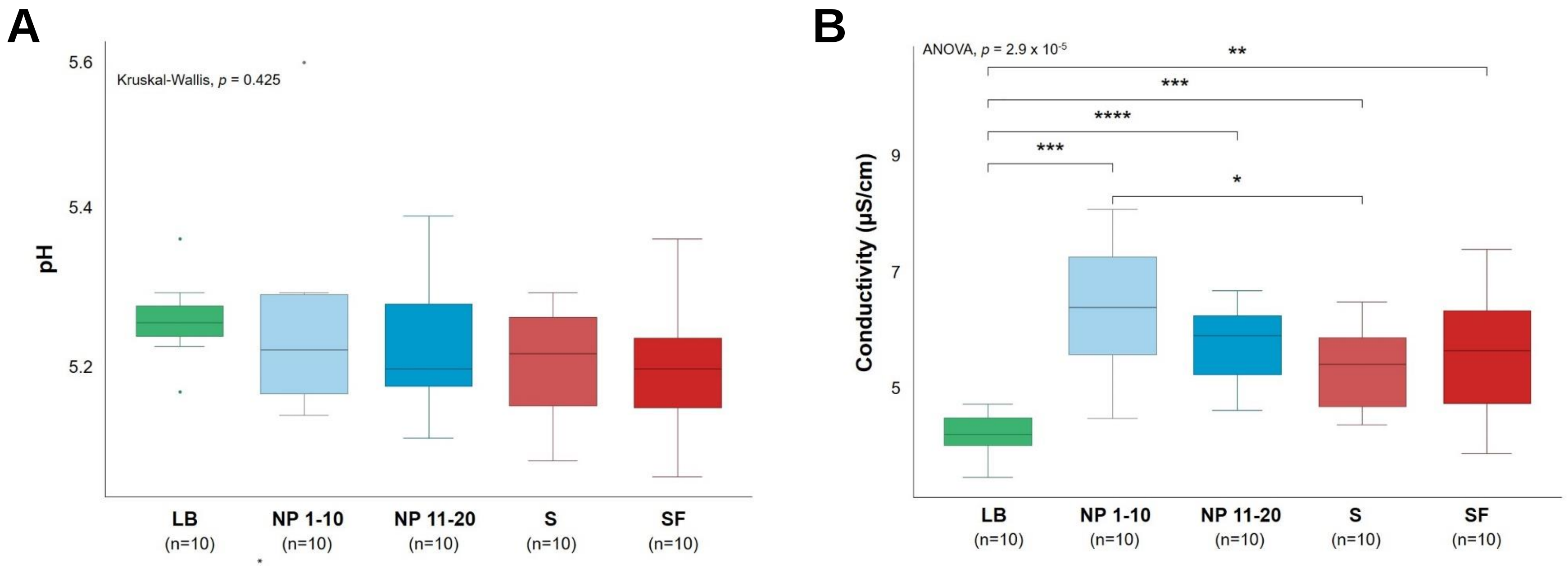

Figure S5. A) pH and B) conductivity per site. NP 1-10: open spaces from the unaffected catchment. NP 11-20: forested area from the unaffected catchment. S: paths from the catchment with human transit. SF: forest areas from the catchment with human transit. Data normality was checked using the Shapiro-Wilk test (p = 0.003 for pH; p = 0.33 for conductivity). Significant differences in pH between sites were determined by pairwise Wilcoxon signed-tank tests. Significant differences in conductivity between sites were determined by pairwise t-tests. *p-value ≤ 0.05. **p-value ≤ 0.01. *** p-value ≤ 0.001. **** p-value ≤ 0.0001. n=10.

Element	DL [µg/L]	Element	DL [µg/L]	Element	DL [µg/L]	Element	DL [µg/L]
**Al**	1.1163	**Be***	0.0035	**Eu***	0.0010	**Ba**	0.0111
**Ti***	0.0222	**B**	0.0547	**Gd***	0.0025	**Hf***	0.0113
**V**	0.0025	**P***	6.9233	**Tb***	0.0015	**Ta***	0.0130
**Mn**	0.0145	**Ca**	19.7270	**Dy***	0.0015	**W***	0.0223
**Co***	0.0014	**Rb**	0.0123	**Ho***	0.0012	**Os***	0.0023
**Ni**	0.0335	**Sr**	0.0693	**Er***	0.0019	**Pt**	0.0043
**Cu**	0.1098	**Zr**	0.0006	**Tm***	0.0129	**Au***	0.0075
**Zn**	0.0273	**Nb***	0.0148	**Yb***	0.0007	**Tl***	0.0084
**Ga**	0.0130	**Mo**	0.0011	**Lu***	0.0010	**Pb**	0.0220
**Ge***	0.0058	**Pd***	0.0267	**Hg***	0.0087	**Bi***	0.0145
**Cd**	0.0068	**Ag***	0.0745	**Na**	2.2319	**Th***	0.0192
**La**	0.0133	**In***	0.0058	**Mg**	3.6128	**U***	0.0130
**Ce**	0.0020	**Sn***	0.0207	**K**	4.8286	**Cr***	0.1018
**Pr***	0.0011	**Sb***	0.0239	**Fe**	0.6113	**As**	0.0033
**Nd***	0.0039	**Te***	0.0037	**Li***	0.0091	**Se***	0.0642
**Sm***	0.0017	**Cs***	0.0070				

Table S2. Detection limit of trace element concentration in melted snow. *More than 25% of the concentration measurements in snow were under the detection limit shown in this table. This trace elements were excluded from further analyses. Three-sigma limit criteria were used. n=10.

Site	NP 11-20 (n=10)			NP 1-10 (n=10)			LB (n=10)			S (n=10)			SF (n=9)		
**Var.**	**Med.**	**Q1**	**Q2**	**Med.**	**Q1**	**Q2**	**Med.**	**Q1**	**Q2**	**Med.**	**Q1**	**Q2**	**Med.**	**Q1**	**Q2**
**Al**	0.0	0.0	0.0	0.0	0.0	0.1	0.0	0.0	0.0	0.0	0.0	0.0	0.0	0.0	0.0
**V**	0.5	0.4	0.7	0.6	0.4	0.6	0.4	0.3	0.4	0.4	0.4	0.5	0.6	0.5	0.9
**Mn**	2.0	1.5	2.8	1.4	1.3	1.5	0.6	0.6	0.8	2.1	1.1	3.3	6.1	1.4	6.9
**Ni**	11.2	8.5	15.1	11.4	10.1	12.3	23.8	17.5	26.6	9.8	7.9	13.1	12.4	11.2	14.5
**Cu**	20.0	10.4	32.1	12.4	9.7	19.3	50.0	38.7	71.1	10.4	6.7	13.6	29.7	15.4	33.6
**Zn**	30.4	28.0	54.8	39.8	32.3	49.9	23.8	13.2	33.9	27.9	22.2	45.1	27.7	21.1	31.5
**Ga**	1.8	1.5	2.1	1.5	1.3	1.7	1.8	1.3	2.2	1.4	1.1	1.7	1.5	1.4	1.6
**Cd**	324.8	265.7	395.1	381.2	327.5	445.7	199.2	129.9	316.7	187.4	166.7	241.6	242.9	183.8	312.3
**La**	1.2	0.7	1.8	1.3	1.0	1.9	0.4	0.3	0.7	1.2	1.0	1.6	1.4	1.2	2.3
**Ce**	0.0	0.0	0.1	0.1	0.0	0.1	0.0	0.0	0.0	0.1	0.1	0.1	0.1	0.1	0.1
**Na**	9.1	5.0	11.5	7.1	6.6	9.0	10.9	8.3	12.1	8.0	4.9	9.2	11.3	7.6	12.3
**Mg**	1.9	1.7	2.2	1.9	1.7	2.2	1.6	1.3	2.1	1.6	1.0	2.0	2.7	1.3	3.7
**K**	3.2	2.1	4.5	3.1	2.6	4.1	1.6	1.2	2.6	2.5	1.6	4.4	6.2	2.7	8.1
**B**	54.2	33.3	69.5	97.7	65.8	126.7	37.0	30.6	46.7	54.2	32.4	61.7	81.5	38.1	110.0
**Ca**	4.4	3.7	4.7	5.3	4.5	7.5	2.9	2.1	5.3	3.8	3.4	4.0	5.1	3.5	5.3
**Rb**	2.0	0.9	2.6	1.0	0.9	1.4	0.3	0.2	0.5	1.6	0.6	5.4	9.6	2.0	12.9
**Sr**	0.8	0.7	1.0	1.0	0.8	1.2	0.6	0.5	0.8	0.9	0.8	1.0	1.1	0.5	1.2
**Mo**	54.6	29.2	79.1	46.6	39.3	59.4	105.3	56.9	152.5	34.8	25.0	58.4	45.3	35.7	57.4
**Ba**	1.0	1.0	1.0	1.0	1.0	1.0	1.0	1.0	1.0	1.0	1.0	1.0	1.0	1.0	1.0
**Pt**	32.6	23.2	41.5	29.7	23.0	42.5	34.3	25.3	37.7	22.8	17.6	26.0	31.2	27.2	44.3
**Pb**	13.6	10.7	24.4	20.2	18.1	27.7	10.1	7.6	15.9	9.1	6.0	14.7	12.3	10.8	14.0
**As**	12.4	4.9	14.5	17.5	15.0	22.8	12.5	9.3	18.2	8.7	7.8	12.5	18.7	11.3	24.7
**Zr**	0.01	0.01	0.01	0.02	0.01	0.02	0.00	0.00	0.01	0.01	0.01	0.01	0.01	0.00	0.01

Table S3. Enrichment factors for selected trace elements in melted snow. NP 1-10: open spaces from the unaffected catchment. NP 11-20: forested area from the unaffected catchment. S: paths from the catchment with human transit. SF: forest areas from the catchment with human transit. Trace elements for which more than 25% of the samples were below detection limit were excluded from the analysis.

Mn		NP1-10	NP11-20	S	SF	LB
	NP1-10		1.00	1.00	0.24	0.91
	NP11-20	1.00		1.00	1.00	0.05
	S	1.00	1.00		1.00	0.04
	SF	0.24	1.00	1.00		0.00
	LB	0.91	0.05	0.04	0.00	

Ce		NP1-10	NP11-20	S	SF	LB
	NP1-10		1.00	1.00	1.00	1.00
	NP11-20	1.00		0.49	1.00	1.00
	S	1.00	0.49		1.00	0.02
	SF	1.00	1.00	1.00		0.23
	LB	1.00	1.00	0.02	0.23	

Ni		NP1-10	NP11-20	S	SF	LB
	NP1-10		1.00	1.00	1.00	0.01
	NP11-20	1.00		1.00	1.00	0.02
	S	1.00	1.00		1.00	0.00
	SF	1.00	1.00	1.00		0.19
	LB	0.01	0.02	0.00	0.19	

Rb		NP1-10	NP11-20	S	SF	LB
	NP1-10		1.00	1.00	0.61	0.25
	NP11-20	1.00		1.00	1.00	0.03
	S	1.00	1.00		1.00	0.04
	SF	0.61	1.00	1.00		0.00
	LB	0.25	0.03	0.04	0.00	

V		NP1-10	NP11-20	S	SF	LB
	NP1-10		1.00	1.00	1.00	0.54
	NP11-20	1.00		1.00	1.00	0.77
	S	1.00	1.00		0.36	1.00
	SF	1.00	1.00	0.36		0.04
	LB	0.54	0.77	1.00	0.04	

K		NP1-10	NP11-20	S	SF	LB
	NP1-10		1.00	1.00	1.00	0.91
	NP11-20	1.00		1.00	1.00	0.65
	S	1.00	1.00		0.97	1.00
	SF	1.00	1.00	0.97		0.02
	LB	0.91	0.65	1.00	0.02	

Cu		NP1-10	NP11-20	S	SF	LB
	NP1-10		1.00	1.00	1.00	0.02
	NP11-20	1.00		0.97	1.00	0.28
	S	1.00	0.97		0.23	0.00
	SF	1.00	1.00	0.23		1.00
	LB	0.02	0.28	0.00	1.00	

Zr		NP1-10	NP11-20	S	SF	LB
	NP1-10		0.97	0.13	0.76	0.01
	NP11-20	0.97		1.00	1.00	0.67
	S	0.13	1.00		1.00	1.00
	SF	0.76	1.00	1.00		1.00
	LB	0.01	0.67	1.00	1.00	

La		NP1-10	NP11-20	S	SF	LB
	NP1-10		1.00	1.00	1.00	0.02
	NP11-20	1.00		1.00	1.00	0.11
	S	1.00	1.00		1.00	0.06
	SF	1.00	1.00	1.00		0.00
	LB	0.02	0.11	0.06	0.00	

B		NP1-10	NP11-20	S	SF	LB
	NP1-10		0.35	0.21	1.00	0.02
	NP11-20	0.35		1.00	1.00	1.00
	S	0.21	1.00		1.00	1.00
	SF	1.00	1.00	1.00		0.21
	LB	0.02	1.00	1.00	0.21	

Cd	Site	NP1-10	NP11-20	S	SF	LB
	NP1-10		1.00	0.03	0.18	0.20
	NP11-20	1.00		0.28	1.00	1.00
	S	0.03	0.28		1.00	1.00
	SF	0.18	1.00	1.00		1.00
	LB	0.20	1.00	1.00	1.00	

Table S4. Kruskal-Wallis comparisons of trace element Enrichment Factors (EF) between sites. NP 1-10: open spaces from the unaffected catchment. NP 11-20: forested area from the unaffected catchment. S: paths from the catchment with human transit. SF: forest areas from the catchment with human transit. Red values indicate significant differences between sites (p-value < 0.05).

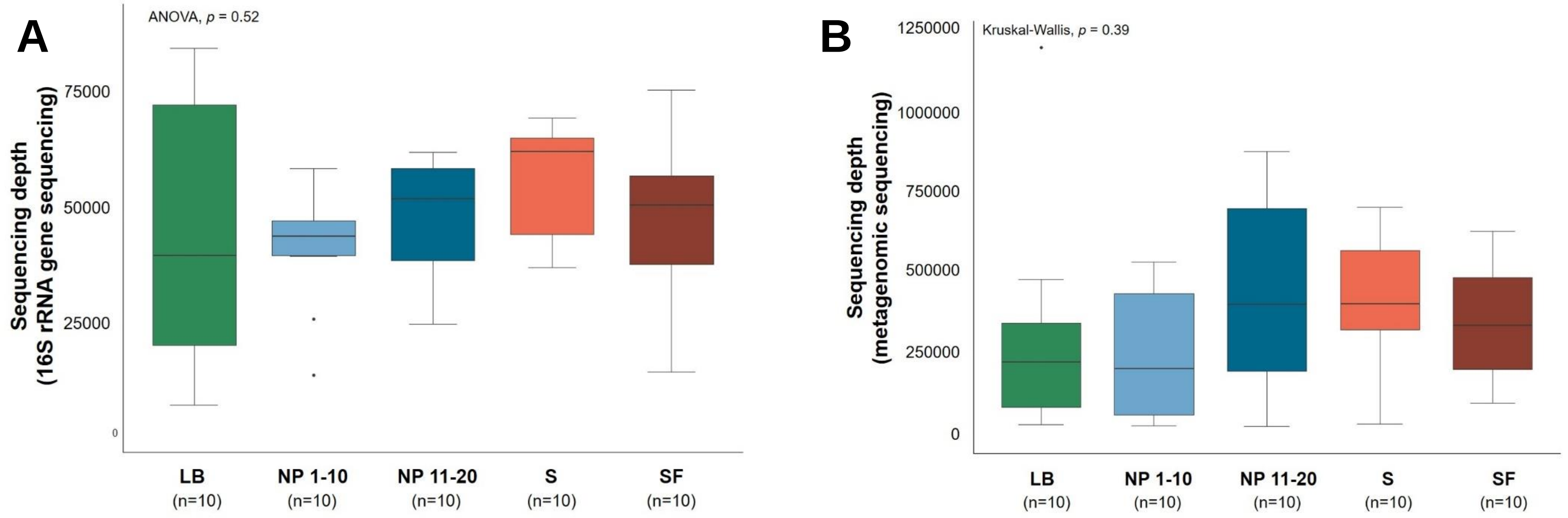

Figure S6. Depths obtained from the sequencing of A) the V3-V4 region of the 16S rRNA gene and B) snow metagenomes. NP 1-10: open spaces from the unaffected catchment. NP 11-20: forested area from the unaffected catchment. S: paths from the catchment with human transit. SF: forest areas from the catchment with human transit. Data normality was checked using the Shapiro-Wilk test (p = 0.003 for pH; p = 0.33 for conductivity). Significant differences in pH between sites were determined by pairwise Wilcoxon signed-tank tests. Significant differences in conductivity between sites were determined by pairwise t-tests. No significant differences were found between sites. n=10.

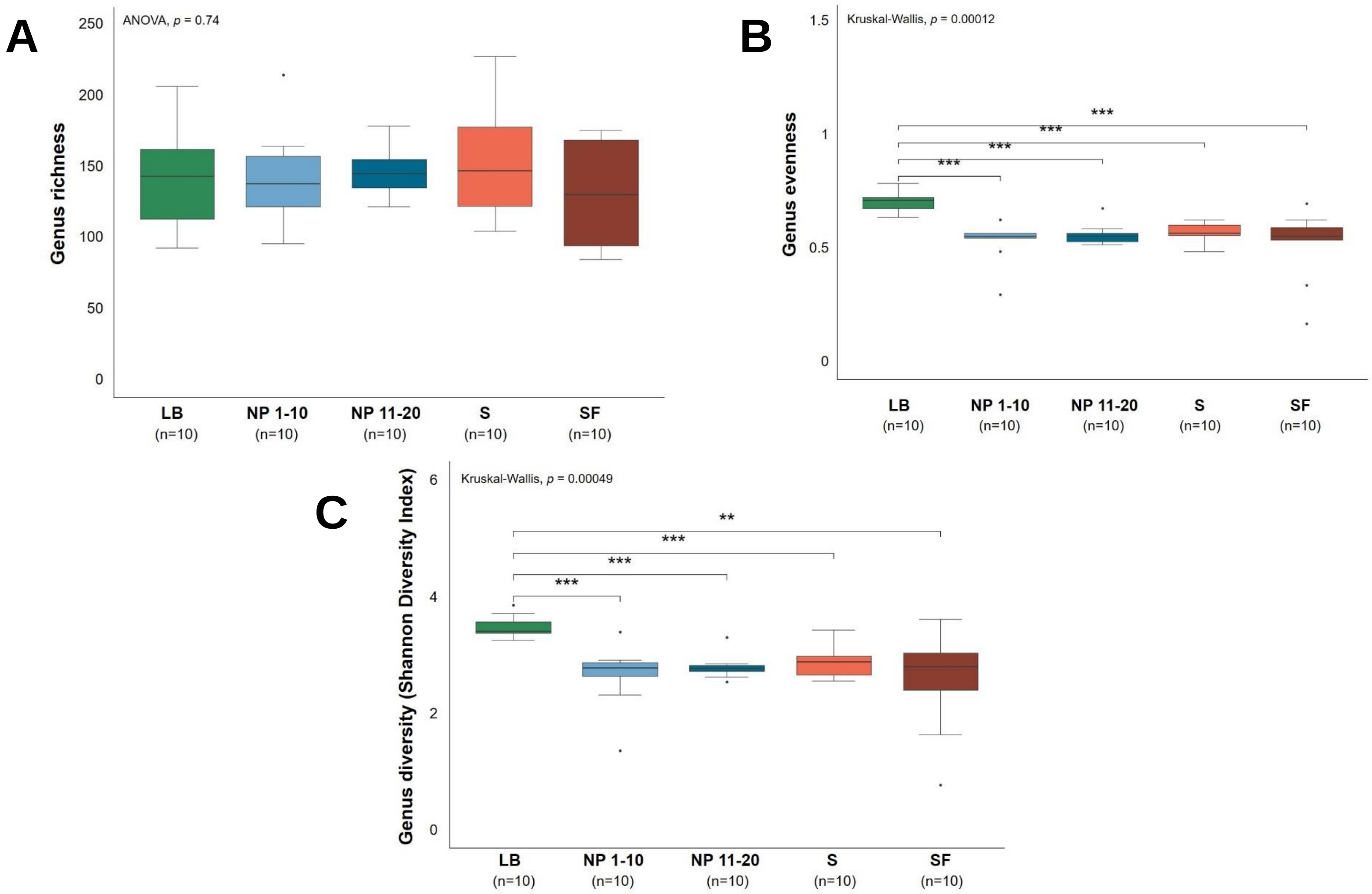

Figure S7. Genus A) richness, B) evenness, C) diversity calculated using the Shannon Index per site. NP 1-10: open spaces from the unaffected catchment. NP 11-20: forested area from the unaffected catchment. S: paths from the catchment with human transit. SF: forest areas from the catchment with human transit. Data normality was checked using the Shapiro-Wilk test (richness: p = 0.47; evenness: p = 2.8 x $^{10-5}$, diversity: p = 0.0001). Significant differences in richness between sites were determined by pairwise t-tests. Significant differences in evenness and diversity between sites were determined by pairwise Wilcoxon signed-tank tests. ***p*-value ≤ 0.01. *** *p*-value ≤ 0.001. n=10.

Physiochemical factor	R^2	Pr (>r)
**NDVI**	0.36	**0.001**
**pH**	0.17	0.195
**Conductivity (µS/cm)**	0.39	**0.001**
**[Mn]**	0.28	**0.002**
**[Ni]**	0.4	**0.001**
**[Cu]**	0.26	**0.006**
**[Cd]**	0.075	0.167
**[B]**	0.24	**0.003**

Table S5. Significance of the correlation between physiochemical parameters and bacterial community composition in snow. Calculated using the envfit function in R with 999 permutations. Red values indicate significant differences between sites (p-value < 0.05).

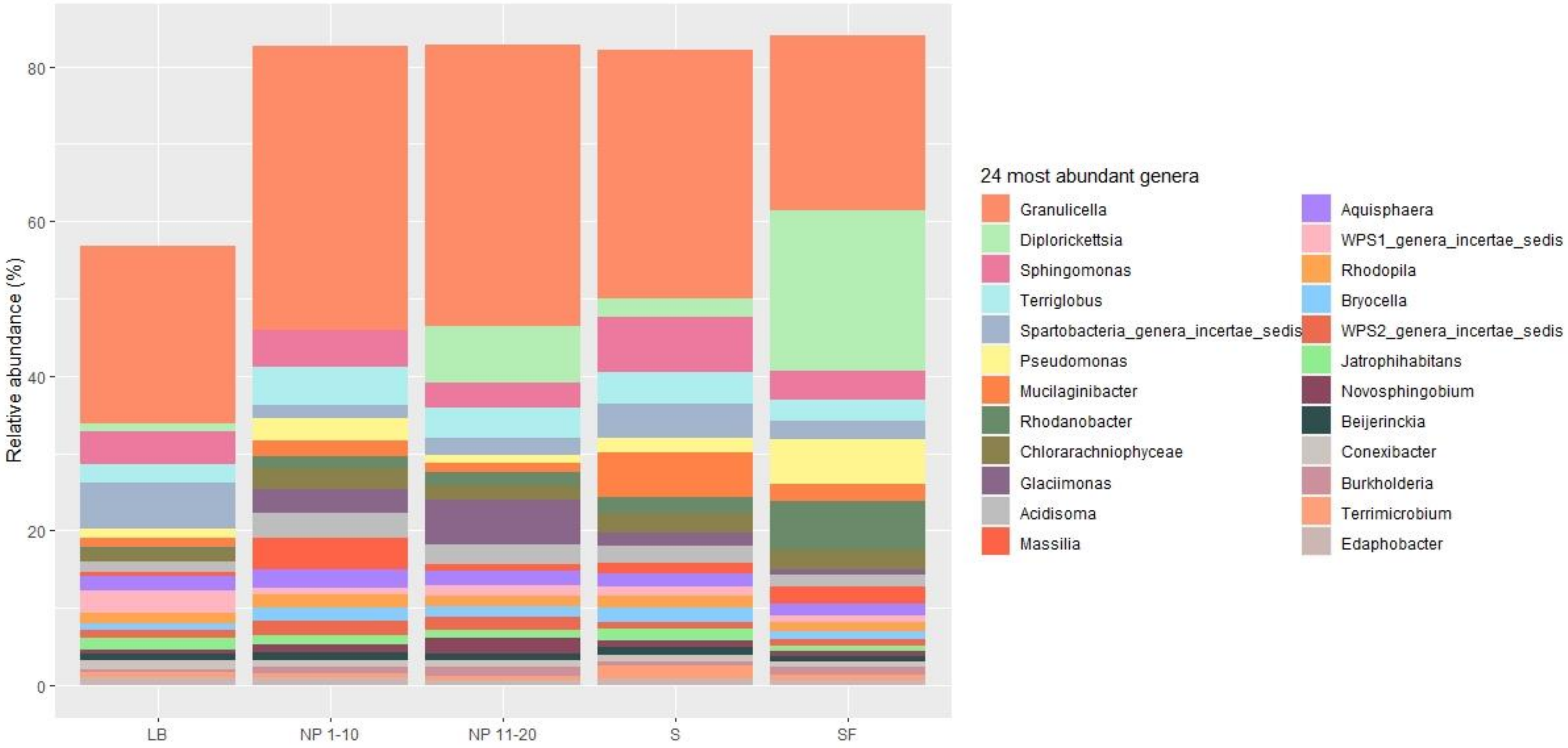

Figure S8. Average relative abundance of the 24 most abundant genera found in snow samples. NP 1-10: open spaces from the unaffected catchment. NP 11-20: forested area from the unaffected catchment. S: paths from the catchment with human transit. SF: forest areas from the catchment with human transit. n=10.

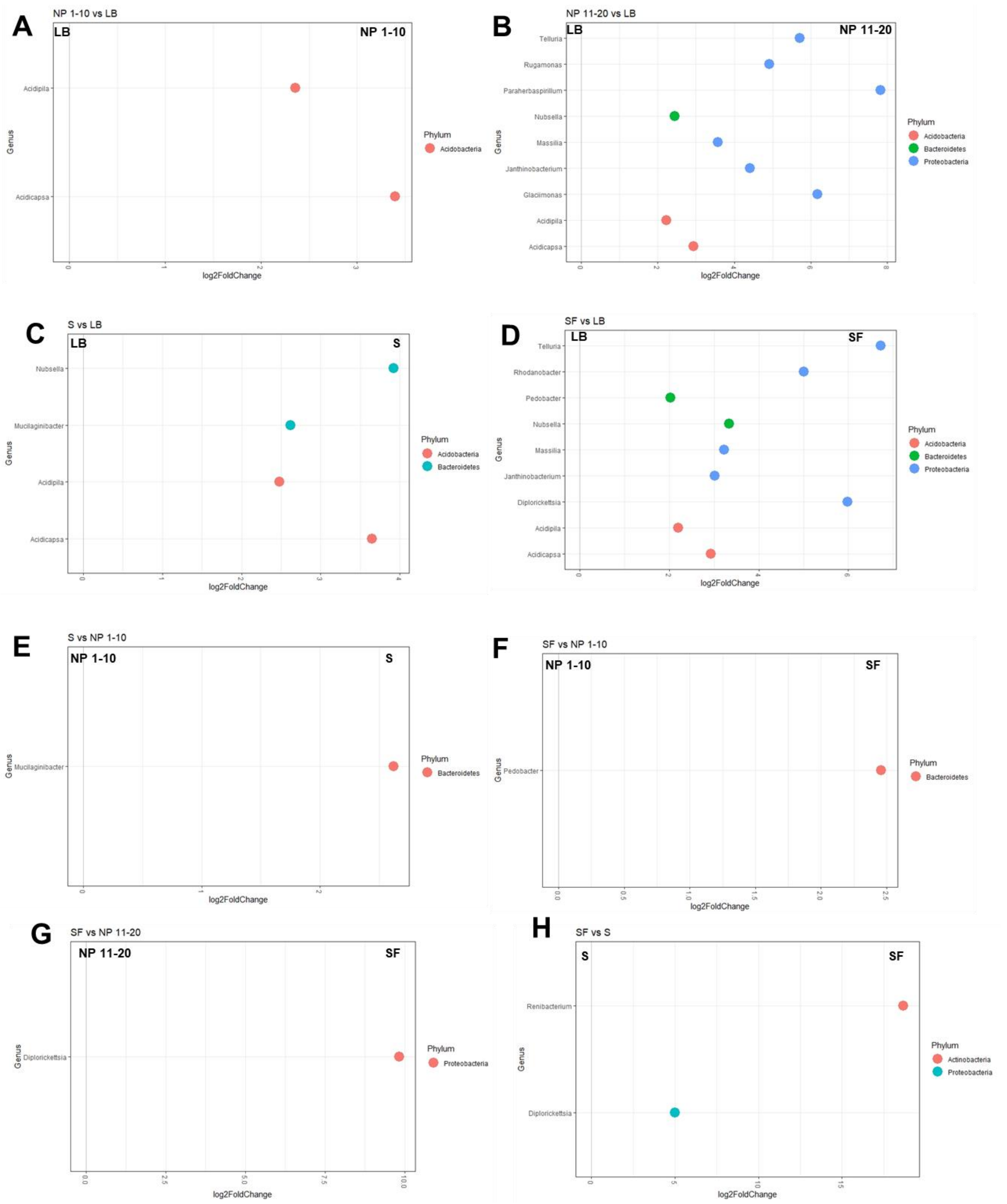

Figure S9. Genus abundance pairwise comparisons between sites. (A) NP 1-10 versus LB; (B) NP 11-20 versus LB; (C) S versus LB; (D) SF versus LB; (E) S versus NP 1-10; (F) SF versus NP 1-10; (G) SF versus NP 11-20; (H) SF versus S. No significant differences were found between NP 1-10 and NP 11-20 nor between S and NP 11-20. NP 1-10: open spaces from the unaffected catchment. NP 11-20: forested area from the unaffected catchment. S: paths from the catchment with human transit. SF: forest areas from the catchment with human transit. Only results with a log2FoldChange ± 2 and an adjusted p-value lower than 0.05 are shown. n=10.

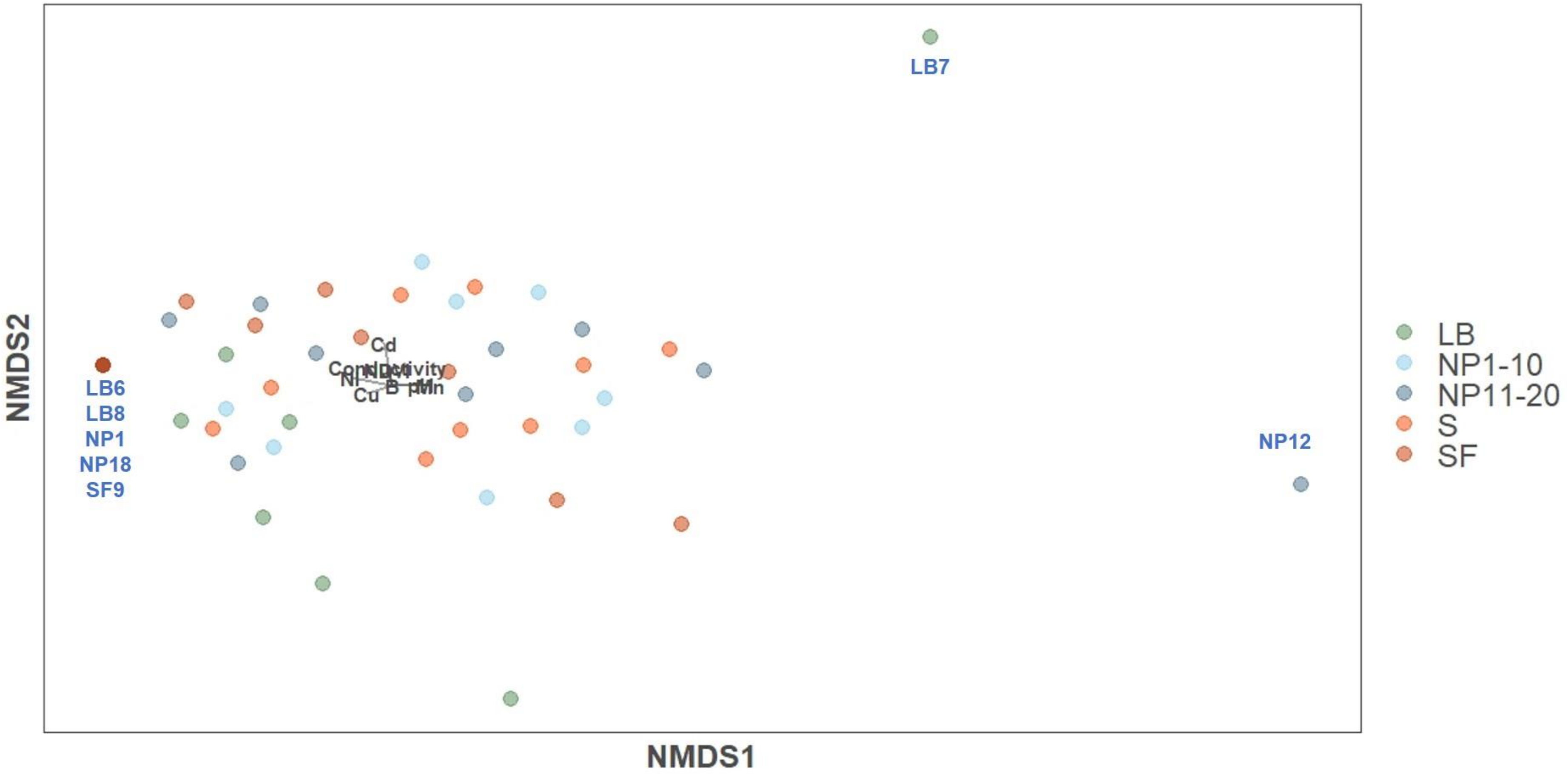

Figure S10. Impact of physiochemical factors on antibiotic resistome composition (NMDS analysis based on Bray-Curtis distances) in snow from all sites. NMDS stress = 0.207. LB: ridge from the catchment with human transit. NP 1-10: open spaces from the unaffected catchment. NP 11-20: forested area from the unaffected catchment. S: paths from the catchment with human transit. SF: forest areas from the catchment with human transit.

Physiochemical factor	R^2	Pr (>r)
**NDVI**	0.0087	0.811
**pH**	0.0086	0.811
**Conductivity (µS/cm)**	0.0125	0.747
**[Mn]**	0.0147	0.731
**[Ni]**	0.0184	0.617
**[Cu]**	0.009	0.79
**[Cd]**	0.0697	0.221
**[B]**	0	0.999

Table S6. Significance of the correlation between physiochemical parameters and antibiotic resistome composition in snow. Calculated using the envfit function in R with 999 permutations. No significant impact was found for any of the physiochemical parameters.

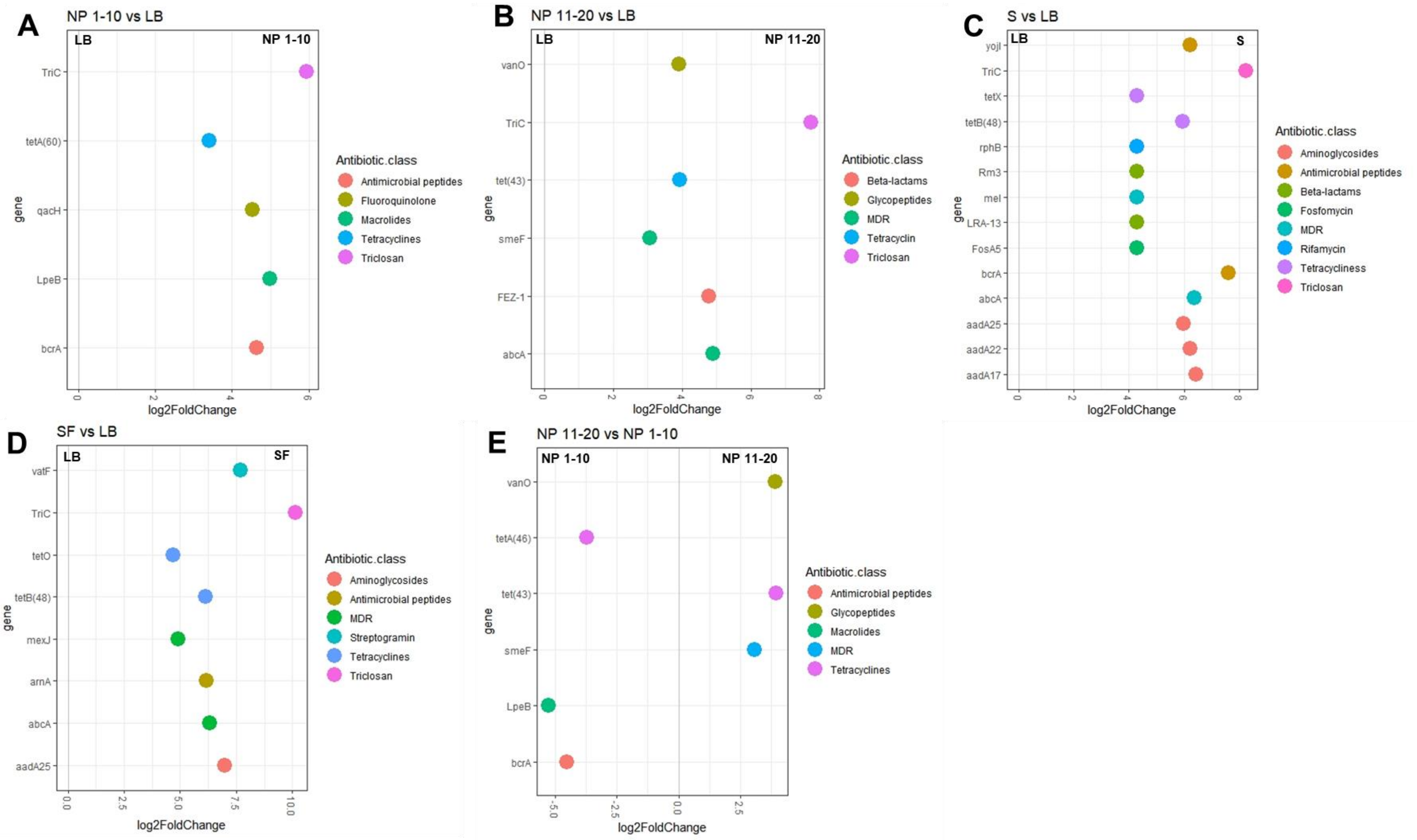

Figure S11. ARG abundance pairwise comparisons between sites that showed significant difference in vegetation levels. (A) NP 1-10 versus LB; (B) NP 11-20 versus LB; (C) S versus LB; (D) SF versus LB; (E) NP 11-20 versus NP 1-10. NP 1-10: open spaces from the unaffected catchment. NP 11-20: forested area from the unaffected catchment. S: paths from the catchment with human transit. SF: forest areas from the catchment with human transit. Only results with a log2FoldChange ± 2 and an adjusted p-value lower than 0.001 are shown. n=10.
